# ZSCAN10 deficiency causes a neurodevelopmental disorder with characteristic oto-facial malformations

**DOI:** 10.1093/brain/awae058

**Published:** 2024-02-22

**Authors:** Lucia Laugwitz, Fubo Cheng, Stephan C Collins, Alexander Hustinx, Nicolas Navarro, Simon Welsch, Helen Cox, Tzung-Chien Hsieh, Aswinkumar Vijayananth, Rebecca Buchert, Benjamin Bender, Stephanie Efthymiou, David Murphy, Faisal Zafar, Nuzhat Rana, Ute Grasshoff, Ruth J Falb, Mona Grimmel, Annette Seibt, Wenxu Zheng, Hamid Ghaedi, Marie Thirion, Sébastien Couette, Reza Azizimalamiri, Saeid Sadeghian, Hamid Galehdari, Mina Zamani, Jawaher Zeighami, Alireza Sedaghat, Samira Molaei Ramshe, Ali Zare, Behnam Alipoor, Dirk Klee, Marc Sturm, Stephan Ossowski, Henry Houlden, Olaf Riess, Dagmar Wieczorek, Ryan Gavin, Reza Maroofian, Peter Krawitz, Binnaz Yalcin, Felix Distelmaier, Tobias B Haack

**Affiliations:** Institute of Medical Genetics and Applied Genomics, University of Tuebingen, Tübingen, 72076, Germany; Department of Neuropediatrics, Developmental Neurology and Social Pediatrics, University of Tübingen, Tübingen 72076, Germany; Institute of Medical Genetics and Applied Genomics, University of Tuebingen, Tübingen, 72076, Germany; Inserm UMR1231, Université de Bourgogne, Dijon Cedex 21070, France; Institute for Genomic Statistics and Bioinformatics, University Hospital Bonn, Rheinische Friedrich-Wilhelms-Universität Bonn, Bonn 53127, Germany; Biogeosciences, UMR 6282 CNRS, EPHE, Université de Bourgogne, Dijon 2100, France; EPHE, PSL University, Paris 75014, France; Department of General Pediatrics, Neonatology and Pediatric Cardiology, Medical Faculty, Heinrich-Heine-University, Düsseldorf 40225, Germany; West Midlands Regional Clinical Genetics Service and Birmingham Health Partners, Birmingham Women’s and Children’s Hospitals NHS Foundation Trust, Birmingham B15 2TG, UK; Institute for Genomic Statistics and Bioinformatics, University Hospital Bonn, Rheinische Friedrich-Wilhelms-Universität Bonn, Bonn 53127, Germany; Institute for Genomic Statistics and Bioinformatics, University Hospital Bonn, Rheinische Friedrich-Wilhelms-Universität Bonn, Bonn 53127, Germany; Institute of Medical Genetics and Applied Genomics, University of Tuebingen, Tübingen, 72076, Germany; Diagnostic and Interventional Neuroradiology, Radiologic Clinics, University of Tübingen, Tübingen 72076, Germany; Department of Neuromuscular Disorders, UCL Queen Square Institute of Neurology, London WC1N 3BG, UK; Department of Clinical and Movement Neurosciences, UCL Queen Square Institute of Neurology, University College London, London WC1N 3BG, UK; Pediatric Neurology, Children’s Hospital, Multan 60000, Pakistan; Pediatric Neurology, Children’s Hospital, Multan 60000, Pakistan; Institute of Medical Genetics and Applied Genomics, University of Tuebingen, Tübingen, 72076, Germany; Center for Rare Disease, University of Tübingen, Tübingen 72072, Germany; Institute of Medical Genetics and Applied Genomics, University of Tuebingen, Tübingen, 72076, Germany; Institute of Medical Genetics and Applied Genomics, University of Tuebingen, Tübingen, 72076, Germany; Department of General Pediatrics, Neonatology and Pediatric Cardiology, Medical Faculty, Heinrich-Heine-University, Düsseldorf 40225, Germany; Institute of Medical Genetics and Applied Genomics, University of Tuebingen, Tübingen, 72076, Germany; Department of Medical Genetics, School of Medicine, Shahid Beheshti University of Medical Sciences, Tehran 1985717443, Iran; Inserm UMR1231, Université de Bourgogne, Dijon Cedex 21070, France; Biogeosciences, UMR 6282 CNRS, EPHE, Université de Bourgogne, Dijon 2100, France; EPHE, PSL University, Paris 75014, France; Department of Pediatric Neurology, Golestan Medical, Educational, and Research Center, Ahvaz Jundishapur University of Medical Sciences, Ahvaz 6135715794, Iran; Department of Pediatric Neurology, Golestan Medical, Educational, and Research Center, Ahvaz Jundishapur University of Medical Sciences, Ahvaz 6135715794, Iran; Department of Biology, Faculty of Science, Shahid Chamran University of Ahvaz, Ahvaz 6135783151, Iran; Department of Biology, Faculty of Science, Shahid Chamran University of Ahvaz, Ahvaz 6135783151, Iran; Narges Medical Genetics and Prenatal Diagnosis Laboratory, Kianpars, Ahvaz 6155689467, Iran; Narges Medical Genetics and Prenatal Diagnosis Laboratory, Kianpars, Ahvaz 6155689467, Iran; Narges Medical Genetics and Prenatal Diagnosis Laboratory, Kianpars, Ahvaz 6155689467, Iran; Diabetes Research Center, Health Research Institute, Ahvaz Jundishapur University of Medical Sciences, Ahvaz 6135715794, Iran; Department of Medical Genetics, School of Medicine, Shahid Beheshti University of Medical Sciences, Tehran 1985717443, Iran; Department of Medical Genetics, School of Medicine, Shahid Beheshti University of Medical Sciences, Tehran 1985717443, Iran; Department of Laboratory Sciences, Faculty of Paramedicine, Yasuj University of Medical Sciences, Yasuj 7591741417, Iran; Department of Pediatric Radiology, Medical Faculty, Institute of Radiology, Heinrich-Heine-University, Düsseldorf 40225, Germany; Institute of Medical Genetics and Applied Genomics, University of Tuebingen, Tübingen, 72076, Germany; Genomics England, Queen Mary University of London, London EC1M 6BQ, UK; Institute of Medical Genetics and Applied Genomics, University of Tuebingen, Tübingen, 72076, Germany; NGS Competence Center Tübingen (NCCT), University of Tübingen, Tübingen 72076, Germany; Department of Neuromuscular Disorders, UCL Queen Square Institute of Neurology, London WC1N 3BG, UK; Institute of Medical Genetics and Applied Genomics, University of Tuebingen, Tübingen, 72076, Germany; Center for Rare Disease, University of Tübingen, Tübingen 72072, Germany; Medical Faculty and University Hospital Düsseldorf, Institute of Human Genetics, Heinrich-Heine-University Düsseldorf, Düsseldorf 40225, Germany; West Midlands Regional Genetics Laboratory, Central and South Genomic Laboratory Hub, Birmingham B15 2TG, UK; Department of Neuromuscular Disorders, UCL Queen Square Institute of Neurology, London WC1N 3BG, UK; Institute for Genomic Statistics and Bioinformatics, University Hospital Bonn, Rheinische Friedrich-Wilhelms-Universität Bonn, Bonn 53127, Germany; Inserm UMR1231, Université de Bourgogne, Dijon Cedex 21070, France; Department of General Pediatrics, Neonatology and Pediatric Cardiology, Medical Faculty, Heinrich-Heine-University, Düsseldorf 40225, Germany; Institute of Medical Genetics and Applied Genomics, University of Tuebingen, Tübingen, 72076, Germany; Center for Rare Disease, University of Tübingen, Tübingen 72072, Germany

**Keywords:** neurodevelopmental disorders, zinc finger transcription factor, oto-facial syndrome, semicircular canal dysplasia

## Abstract

Neurodevelopmental disorders are major indications for genetic referral and have been linked to more than 1500 loci including genes encoding transcriptional regulators. The dysfunction of transcription factors often results in characteristic syndromic presentations; however, at least half of these patients lack a genetic diagnosis. The implementation of machine learning approaches has the potential to aid in the identification of new disease genes and delineate associated phenotypes.

Next generation sequencing was performed in seven affected individuals with neurodevelopmental delay and dysmorphic features. Clinical characterization included reanalysis of available neuroimaging datasets and 2D portrait image analysis with GestaltMatcher. The functional consequences of ZSCAN10 loss were modelled in mouse embryonic stem cells (mESCs), including a knockout and a representative *ZSCAN10* protein truncating variant. These models were characterized by gene expression and western blot analyses, chromatin immunoprecipitation and quantitative PCR (ChIP-qPCR) and immunofluorescence staining. *Zscan10* knockout mouse embryos were generated and phenotyped.

We prioritized bi-allelic *ZSCAN10* loss-of-function variants in seven affected individuals from five unrelated families as the underlying molecular cause. RNA-sequencing analyses in *Zscan10*^−/−^ mESCs indicated dysregulation of genes related to stem cell pluripotency. In addition, we established in mESCs the loss-of-function mechanism for a representative human *ZSCAN10* protein truncating variant by showing alteration of its expression levels and subcellular localization, interfering with its binding to DNA enhancer targets. Deep phenotyping revealed global developmental delay, facial asymmetry and malformations of the outer ear as consistent clinical features. Cerebral MRI showed dysplasia of the semicircular canals as an anatomical correlate of sensorineural hearing loss. Facial asymmetry was confirmed as a clinical feature by GestaltMatcher and was recapitulated in the *Zscan10* mouse model along with inner and outer ear malformations.

Our findings provide evidence of a novel syndromic neurodevelopmental disorder caused by bi-allelic loss-of-function variants in *ZSCAN10*.

## Teaser

Laugwitz *et al.* describe a novel syndromic neurodevelopmental disorder with characteristic malformations of the face and of the inner and outer ear. The autosomal recessive disorder is caused by biallelic loss-of-function variants in the *ZSCAN10* gene.

## Introduction

Major congenital anomalies are observed in 2%–5% of live births and are often accompanied by developmental delay (DD) and intellectual disability (ID).^1^ These syndromic forms of neurodevelopmental disorders are among the most common indications for genetic referral and represent a heterogenous group of conditions that can severely affect a child’s development. To date, more than 1500 loci have been linked to syndromic and non-syndromic forms of DD/ID, which collectively have a global prevalence of approximately 1%.^2,3^ While diverse mechanisms can cause neurodevelopmental disorders, pathogenic genetic variation in developmentally important genes has a major contribution.^4,5^ Some of these genes encode transcriptional regulators, and individuals affected with these disease entities often present with numerous clinical features in addition to DD/ID such as organ malformations and facial abnormalities.^6^

**Figure 1:**
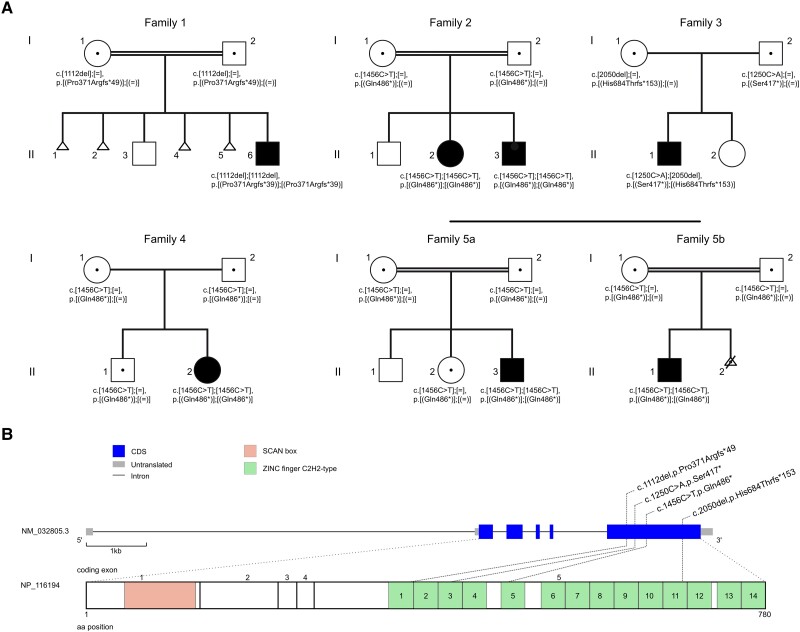
**Pedigrees of investigated families and structure of *ZSCAN10.*** (**A**) Pedigrees of five families with pathogenic variants in *ZSCAN10*, illustrating affected (filled symbol), healthy (open symbol) family members. Heterozygous carriers are indicated (open symbols containing central dot). Unaffected siblings and fetuses were not tested unless indicated. Families F5a and F5b were remotely related. Individual F5b:II.1 exhibited thalassaemia minor as an additional phenotype. Individual F5b.II.2 was intentionally aborted due to genetically confirmed thalassaemia major (asterisk); the fetus was a carrier of the c.1456C>T variant in *ZSCAN10*. (**B**) Structure of *ZSCAN10* and the encoded protein with known domains and position of identified variants. aa = amino acid; CDS = coding sequence.

The largest human transcription factor family, the zinc finger proteins, contains >800 members, several of which are associated with syndromic neurodevelopmental disorders, e.g. *GLI3*: OMIM #175700; *ZIC1*: OMIM #618736; and *ZNF462*: OMIM #618619.^7-11^*ZSCAN10* (also known as *ZFP206*: OMIM *618365) encodes a human zinc finger-transcription factor of the C_2_H_2_ zinc finger subfamily with an additional N-terminal SCAN domain (Fig. 1).^8,12^ It is expressed in human embryonic stem cells where it is believed to function in the maintenance of pluripotency within the transcriptional network of other transcription factors like OCT4, SOX2 and NANOG.^13-16^ Compatible with a predominant role in early embryonic development *ZSCAN10* mRNA expression decreases with age but remains postnatally detectable in the brain, testis and pituitary gland.^17,18^

Here, we report seven affected individuals from five families with a clinically recognizable form of syndromic DD/ID due to bi-allelic loss-of-function variants in *ZSCAN10* (Fig. 1).

## Materials and methods

### Clinical assessment

Clinical data were provided by the primary physicians. Consent to publish images was obtained from legal guardians. All procedures were performed in accordance with the Declaration of Helsinki (Supplementary material).

### Genetic studies

After obtaining written informed consent, exome or genome sequencing was performed as previously described (Supplementary material).^19,20^ Assembly of this international collaborative study cohort resulted from personal communication between collaborators (Families F4 and F5) and was facilitated by GeneMatcher (Family F3).^21^ Runs of homozygosity were evaluated using GSvar (https://github.com/imgag/ngs-bits/blob/master/doc/GSvar/roh_analysis.md). The possibility of shared ancestry was evaluated based on the available sequencing datasets by manual inspection of genotypes in the genomic region surrounding the *ZSCAN10* locus.

### *Zscan10* knockout in mouse embryonic stem cells

Feeder-free HM1 mouse embryonic stem cells (mESCs) used in this study were previously described.^22^ HM1 mESCs were grown in standard medium [Dulbecco’s modified Eagle medium supplemented with 15% fetal bovine serum (FBS), ES-qualified, (ThermoFisher Scientific, 16141-079), 1000 U/ml leukemia inhibitory factor (Millipore), 1× Minimum Essential Medium Non-Essential Amino Acids solution, 1 mM of sodium pyruvate, 1× GlutaMAX™ supplement, 1× penicillin and streptomycin and 0.1 mM of 2-mercaptoethanol] supplemented with 1 µM MEK1/2 inhibitor (PD0325901, Sigma) and 3 µM GSK3 inhibitor (CHIR99021, Sigma). Cells were grown on 0.2% gelatinized (Sigma, G1890) tissue culture plates or dishes.

The clustered regularly interspaced short palindromic repeats (CRISPR)/Cas9 system was used to knock out exon 2 of *Zscan10* to generate *Zscan10*^−/−^ mESCs as previously described.^23^ The guide RNAs (gRNAs) were designed using CRISpick (https://portals.broadinstitute.org/gppx/crispick/public) and synthesized by biomers (www.biomers.net). Two guide RNAs targeting the upstream and downstream of *Zscan10* exon2 were picked up and sub-cloned into the pX459 vector (upstream gRNA1: ATACTGCGTTAAGATCTGAC; downstream gRNA2: AGAGGTGAGTGGAAAGAAAC). PCR and Sanger sequencing were used to select the positive cell clones (forward primer: ACTCCTGGTCCTCTAGACTC; reverse primer: CTAGCTATGCTCACTGCCTT) (Supplementary Fig. 1).

### RNA-sequencing and expression analysis

RNA-sequencing (RNA-seq) and data analysis were performed as previously described.^24^ A total of 100 ng total RNA extracted from mESCs was subjected to polyA enrichment and cDNA sequencing libraries were prepared using a NEBNext Ultra II Directional RNA Library Preparation Kit for Illumina (NEB, E7760L). Libraries were sequenced as paired-end reads on a NovaSeq6000 (Illumina) with at least 20 million reads each. After quality control, RNA-seq data were aligned using HISAT2 (version 2.2.1) to Ensemble Mouse GRCm38 (mm10). Normalized read counts for all genes were obtained using featureCount (v3.18.1). Differentially expressed genes [false discovery rate (FDR) < 0.05] were determined in comparisons between experimental and control groups using Differential Expression Analysis for Sequence Count Data (DESeq, v1.22.1).

### Characterization of the impact of the human *ZSCAN10* c.1456C>T variant

The human wild-type *ZSCAN10* cDNA was purchased from Genscript (OHu73812D) and PCR amplified and subcloned into pcDNA3/C-SF vector using HindIII and EcoRI.^23^ The *ZSCAN10* c.1456C>T variant cDNA was obtained using PCR amplification of the first 1455 bp coding region of the full-length *ZSCAN10* cDNA, encoding the first 485 amino acids of the ZSCAN10 protein. The mutant *ZSCAN10* cDNA was subcloned into the pcDNA3/C-SF vector using HindIII and EcoRI. The full-length *ZSCAN10* (ZSCAN10 full) and c.1456C>T *ZSCAN10* (ZSCAN10 485) expression vectors were transfected into mESCs using Lipofectamine 2000 (Invitrogen). Forty-eight hours after transfection, the cells were harvested for western blot analysis, immunofluorescence staining and chromatin immunoprecipitation and quantitative PCR (ChIP-qPCR) analysis (Supplementary material).

### GestaltMatcher analysis

We analysed facial similarities among affected subjects with confirmed genetic disorders harbouring pathogenic variants in string interacting genes including *ZSCAN10*, *CHD7*, *PBX1*, *SALL4* and *SMAD4* using GestaltMatcher.^25,26^ The dataset consisted of 90 frontal images in total. 2D images of five subjects with ZSCAN10 deficiency (Individuals F1:II.6, F2:II.2, F2:II.3, F3:II.1 and F5B:II.1) were available for facial analysis (Supplementary Table 2). GestaltMatcher was first trained on 3438 images with 139 different disorders from the GestaltMatcher database to learn the facial dysmorphic features and later encoded each image into a 320-dimensional facial phenotype descriptor (FPD). The FPDs formed a Clinical Face Phenotype Space, and the facial syndromic similarity was quantified by the cosine distance between two FPDs in the space. We performed t-distributed stochastic neighbour embedding to visualize the 90 FPDs in 2D space and expanded the model to recognize facial asymmetry (Supplementary Table 3).^27^

### Dysmorphism analyses in mouse embryos

We used mice homozygous for a gene targeting *Zscan10* allele generated by homologous recombination in ESCs using the knockout-first allele method,^28^ adopting a strategy identifying exon 6 common to all transcripts of *Zscan10*. This strategy perturbs the expression of the targeted gene by inclusion of a large LacZ cassette in the upstream region of exon 6 that interferes with transcription producing the *Zscan10^tm2a(EUCOMM)Wtsi^* constitutive knockout allele. The mouse model was validated according to standardized quality control procedures (https://www.mousephenotype.org/data/alleles/qc_data/mouse/MDNC/).

Episcopic image stacks were obtained from the Deciphering the Mechanisms of Developmental Disorders (DMDD) consortium for morphometric analysis as described previously.^29,30^ Ten paired landmarks and six unpaired landmarks were digitized three times on the 3D surface of each head surface using the R package digit3DLand (https://github.com/morphOptics/digit3DLand). Morphometric analysis with a focus on facial asymmetry was performed blinded for the genotype using 26 well-defined landmarks on mouse 3D surface meshes including 10 midline structures and 16 lateral/medial structures. For inner ear reconstructions, 22 points were digitized on the manually segmented models. A full generalized Procrustes superimposition with object symmetry was performed using the R package Morpho.^31^ A Procrustes ANOVA for object symmetry was computed to estimate fluctuating asymmetry (FA) and the among-individuals variance.^32,33^ The sum of squares of these variance components was decomposed according to the genotypes. FA was defined according to the following equation: FA = 2(mean square_individual__×__side_ − mean square_error_) (Supplementary material).

## Results

### Genetic characterization

Genetic diagnostic testing failed to identify pathogenic or likely pathogenic variants in established disease genes that have been associated with the affected individuals’ clinical phenotypes. However, a subsequent search for genes carrying ultra-rare, predicted protein-truncating variants (PTVs) prioritized compound heterozygous or homozygous variants in *ZSCAN10* in all seven affected individuals (Fig. 1 and Table 1). No homozygous PTVs in *ZSCAN10* were listed in gnomAD (v2.1.1) or in-house databases. Carrier testing on available family members showed full co-segregation of the *ZSCNA10* variants with the clinical phenotype (Fig. 1).

**Table 1:** Clinical and genetic findings in individuals with bi-allelic ZSCAN10 variants

Individual	F1:II.6	F2:II.2	F2:II.3	F3.II.1	F4:II.2	F5a:II.3	F5b:II.1
Gender	male	female	male	male	female	female	male
Country of origin	Turkey	Iran	Iran	India	Pakistan	Iran	Iran
cDNA change(s)^a^	c.1112del	c.1456C>T	c.1456C>T	c.1250C>A; c.2050del	c.1456C>T	c.1456C>T	c.1456C>T
Protein change(s)^b^	p.Pro371Argfs*49	p.Gln486*	p.Gln486*	p.Ser417*; p.His684Thrfs*153	p.Gln486*	p.Gln486*	p.Gln486*
Allelic status	Homozygous	Homozygous	Homozygous	Cmpd. heterozygous	Homozygous	Homozygous	Homozygous
Age at onset/last examination	Congenital/9 y 6 m	Congenital/15 y	Congenital/11 y	Congenital/1 y 8 m	Congenital/2 y	Neonatal period/3 y 7 m	Congenital/10 y 3 m
Cognitive impairment	Moderate	Moderate, minimal speech development	Mild	Moderate	Severe, no expressive language	Severe	Severe, no expressive language
Delay of motor development	Moderate	Severe	Severe	Moderate	Profound	Moderate	Severe
Behavioural abnormalities	Autistic features, hyperphagia	Aggression	−	Autistic features, aggression	−	Stereotypic movements	−
Vision impairment	+	n.d.	n.d.	−	−	+	+
Hearing impairment	+	n.d.	n.d.	Unilateral deafness	+	−	Profound bilateral SNHL
Other organ systems	Micropenis, NASH	Cleft plate	Cardiac malformation	Micropenis, maldescended testis	−	Mild left ventricular enlargement	Thalassaemia minor
Dysmorphic facial features	Asymmetry	Asymmetry, reduced left facial movement, broad nasal root	Asymmetry, reduced left facial movement, broad nasal root	Asymmetry, reduced left facial movement, down slanting palpebral fissures, prominent epicanthic folds	Large epicanthal folds, high arched palate	−	Hypotonic facies
Outer ear	Unilateral malformation with angulated antihelix, overfolded helix, absent superior crus of antihelix	Bilateral malformation with low set, posteriorly rotated, angulated antihelix, overfolded helix, small ear lobes, absent superior crus of antihelix	Bilateral malformation: posteriorly rotated, left: conchal shelf, right: overfolded helix, small superior crus of antihelix	Unilateral severe dysplasia	Unilateral malformation: microtia, low-set, posteriorly rotated, absent superior crus of antihelix	Bilateral malformation: microtia, low-set, posteriorly rotated, absent superior crus of antihelix	Unilateral malformation: left microtia, dysplasia, low-set, crumpled ear
Inner ear	Bilateral SCCs dysplasia	n.d.	n.d.	Bilateral SCC dysplasia	n.d.	n.d.	n.d.

Compd. compound; m = months; n.d. = not determined; NASH = non-alcoholic steatohepatosis; SCC = semicircular canal; SNHL = sensineural hearing loss; y = years; + = present; − = absent.

^a^GenBank: NM_032805.3.

^b^GenBank: NP_116194.

From the four unique *ZSCAN10* variants identified, only the stop variant c.1456C>T, p.(Gln486*) was listed in gnomAD (v2.1.1; 17 times in a heterozygous state in the South Asian cohort corresponding to a minor allele frequency of 7 × 10^−4^ in this subpopulation.). This change was observed in a homozygous state in five affected individuals from Families F2, F4 and F5, originating from neighbouring geographic regions in Iran and Pakistan. Concordant with the reported consanguinity in Families F2 and F5, we detected extended runs of homozygosity (∼2.5 Mb to ∼8.1 Mb) encompassing *ZSCAN10* and harbouring the same homozygous common variants over a 1.9 Mb region. These data are in line with a distant shared ancestry among these families.

### *In silico* prediction of loss of ZSCAN10 function

Based on *in silico* predictions, loss of ZSCAN10 function is the likely pathomechanism for the identified disease alleles (Fig. 1 and Supplementary Fig. 1). The *ZSCAN10* transcription unit is composed of six exons including a first non-coding exon. The last exon, exon 6, is very large and contains 66% (518 out of 780 amino acids) of the protein, including all 14 C_2_H_2_-type zinc finger motifs. Notably, all identified variants were PTVs located within exon 6, with the most 5′ variant, the 1 bp deletion c.1112del, being located 160 bp downstream of the last exon-exon junction and the most 3′ variant, the 1 bp deletion c.2050del, being located 293 bp upstream of the termination codon. According to the rules governing nonsense-mediated mRNA decay (NMD), the localization of a PTV downstream of the last exon-junction complex (more specifically, no further upstream than 50–55 nucleotides before the last exon-exon junction) and the presence of very long exons lead to reduced NMD efficiency.^34,35^ However, NMD predictions differ between e.g. the most upstream and downstream PTVs identified (NMDEscPredictor; Supplementary Fig. 1), and the question of whether aberrant transcripts escape NMD or not at endogenous expression levels could not be investigated for lack of a suitable model system. In case aberrant *ZSCAN10* mRNAs escape NMD, translation might result in prematurely truncated polypeptides missing 13% to 56% of the C-terminal wild-type protein sequence. This region contains the 14 highly conserved C_2_H_2_-type zinc finger motifs (encoded by amino acids p.347–p.779), and even the most downstream PTV c.2050del, p.(His684Thrfs*153) alters nearly a third of them (motifs 11–14). These considerations together with the autosomal recessive interheritance patterns are in line with the hypothesis that loss of ZSCAN10 function is the likely consequence and pathomechanism of the identified disease alleles (Fig. 1).

### Altered expression of stem cell marker genes in mouse embryonic stem cells due to loss of ZSCAN10

**Figure 2:**
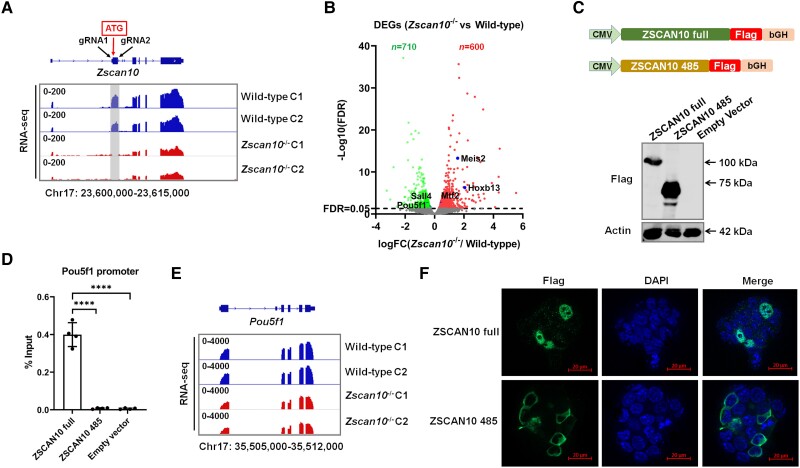
**Characterization of *Zscan10* in mouse endothelial stem cells and its functional impact.** (**A**) *Top*: Schematic diagram showing the strategy to knock out the *Zscan10* gene in mouse endothelial stem cells (mESCs). Two gRNAs targeting upstream (gRNA1) and downstream (gRNA2) of *Zscan10* exon 2 were used to knock out exon 2 of the *Zscan10* gene in mESCs. *Bottom*: RNA-sequencing (RNA-seq) data coverage plot showing the expression of the *Zscan10* gene in wild-type and *Zscan10* knockout mESCs (*Zscan10^−/−^*). Exon 2 of *Zscan10* is lost in *Zscan10*^−/−^ cell lines (grey shaded area). (**B**) Volcano plot showing the differentially expressed genes (DEGs) in *Zscan10*^−/−^ mESCs compared with control mESCs, including 710 downregulated genes and 600 upregulated genes. The genes regulated by Zscan10 and related to pluripotency and differentiation of ESCs are labelled. FC = fold-change; FDR = false discovery rate. (**C**) *Top*: Schematic diagrams showing the structures of wild-type *ZSCAN10* (ZSCAN10 full) and *ZSCAN10* c.1456C>T variant (expressing only the first 485 amino acids of the ZSCAN10 protein) expression vectors. *Bottom*: Representative western blot showing expression of ZSCAN10 full and ZSCAN10 485 protein. (**D**) Chromatin immunoprecipitation and quantitative PCR (ChIP-qPCR) shows the binding affinity of ZSCAN10 full, ZSCAN10 485 mutated protein on the *Pou5f1* promoter in different vector transfected mESCs. Empty vector transfected mESCs were used as a negative control. ****P < 0.0001. (**E**) RNA-seq data coverage plot showing the expression of the *Pou5f1* gene in wild-type and *Zscan10*^−/−^ mESCs. Chr17 = chromosome 17. (**F**) Representative images of immunofluorescence staining show the subcellular distribution of ZSCAN10 full and ZSCAN10 485 proteins. The wild-type ZSCAN10 protein (ZSCAN10 full) is located mainly in the nucleus, whereas the mutant ZSCAN10 protein (ZSCAN10 485) is located mainly in the cytoplasm.

To further model the loss-of-function consequences in embryonic development, we generated *Zscan10*^−/−^ mESCs lines using the CRISPR/Cas9 technique with two gRNAs targeting upstream and downstream sequences of exon 2 in which the translational start site (ATG) is located (Fig. 2A). RNA-seq confirmed loss of exon 2 expression in *Zscan10*^−/−^ mESCs cell lines (Fig. 2A). The expression of other exons was also decreased (Fig. 2A), which might be due to NMD. RNA-seq analysis detected 1310 differentially expressed genes, of which 710 genes were downregulated and 600 genes were upregulated (FDR < 0.05) (Fig. 2B). KEGG pathway analysis revealed that the significantly affected pathway of the downregulated genes is ‘ATP-dependent chromatin remodeling’, while the significantly affected pathway of the upregulated genes is ‘oxidative phosphorylation’. When comparing the differentially expressed genes with ZSCAN10 target genes in mESCs,^36^ we observed that some of these genes related to stem cell pluripotency and differentiation were dysregulated, including *Pou5f1*, *Sall4*, *Mtf2*, *Hoxb13* and *Meis2* (Fig. 2B).^36^ This observation supports the function of ZSCAN10 in maintaining ESC pluripotency and regulating their differentiation.

### Impact of human *ZSCAN10* protein truncating variant on subcellular distribution and DNA binding

Next, we sought to investigate the mechanism by which the identified variants could affect the biological function of ZSCAN10. We first generated a wild-type human *ZSCAN10* expression vector and a mutant *ZSCAN10* expression (c.1456C>T) vector (Fig. 2C). The *ZSCAN10* c.1456C>T variant was found in many families in our cohort (Fig. 1) and was therefore considered representative of the pathomechanism. Western blot analysis confirmed the expression of both the full-length *ZSCAN10* vector (ZSCAN10 full) and the c.1456C>T variant *ZSCAN10* (ZSCAN10 485) vector (Fig. 2C). As *POU5F1* is a critical target gene of ZSCAN10 (also known as ZFP206),^36^ we next performed ChIP-qPCR to show ZSCAN10 binding on mouse *Pou5f1* gene promoter using an anti-flag antibody and mESCs transfected with different ZSCAN10 vectors. We observed that wild-type ZSCAN10 (ZSCAN10 full) strongly bound to *Pou5f1* promoter but the mutant ZSCAN10 (ZSCAN10 485) lost its binding activity on the *Pou5f1* promoter (Fig. 2D). RNA-seq analysis also confirmed decreased expression of Pou5f1 in *Zscan10*^−/−^ mESCs (Fig. 2E and Supplementary material). In addition, we performed immunofluorescence staining to determine the subcellular distribution of the wild-type and mutant ZSCAN10. This analysis revealed that the wild-type ZSCAN10 (ZSCAN10 full) was mainly localized in nuclei, whereas the mutant ZSCAN10 (ZSCAN10 485) protein was mainly distributed in the cytoplasm. This observation supports the notion that the *ZSCAN10* c.1456C>T variant disrupts the nuclear transportation of the encoded truncated protein, which could also explain why the mutant ZSCAN10 protein has lost DNA binding to the *Pou5f1* promoter. These experimental data further substantiate the predicted detrimental impact of the identified variants and that loss-of-function is the likely pathomechanism underlying *ZSCAN10*-associated disease.

### Clinical phenotype

**Figure 3:**
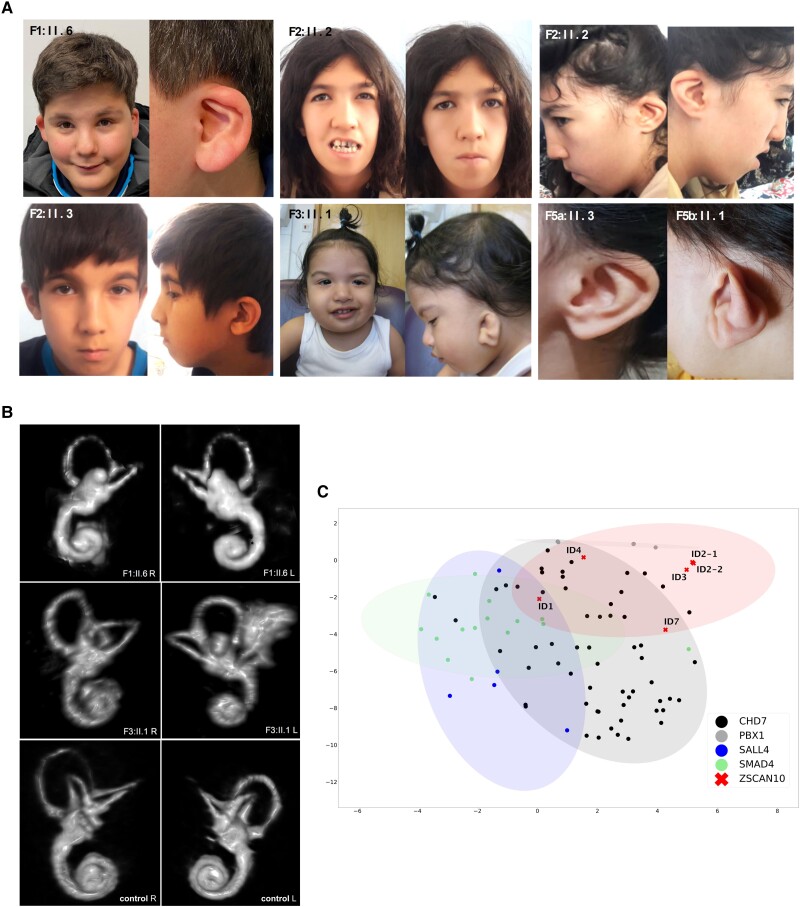
**Facial dysmorphisms and neuroimaging findings in subjects with ZSCAN10 deficiency.** (**A**) Dysmorphic features in ZSCAN10 deficiency include facial asymmetry (Individual F1:II.6 and F3:II.1) with reduced facial movements, outer ear malformations, down slanting palpebral fissures and prominent epicanthic folds. (**B**) MRI-based 3D reconstruction of the inner ear. Right (R) and left (L) inner ear reconstructions showing bilateral aplasia of the horizontal semicircular canals (SCC) and dysplasia of the vestibule in Individual F1:II.6 and SCC dysplasia in Individual F3:II.1 as well as a healthy individual as the control. (**C**) 2D visualization of 90 images from individuals with pathogenic variants in *ZSCAN10*, *CHD7*, *PBX1*, *SALL4* and *SMAD4* by t-distributed stochastic neighbour embedding.

Pregnancy and birth measurements were reportedly normal in all individuals. However, due to variable congenital abnormalities including outer ear (7/7) and inner ear malformations (2/2 MRI datasets available for reanalysis) with variable hearing impairment (4/5), microgenitalia (2/7), heart defects (1/7) and cleft palate (1/7), all individuals underwent further medical examination after birth. Common dysmorphic features noted in most of the affected individuals (5/7) were facial asymmetry with unilaterally reduced facial movements or hypotonic facies, down slanting palpebral fissures and prominent epicanthic folds (Fig. 3). The dysmorphic facial features were highly consistent among the affected individuals, although of variable degrees. In Individuals F1:II.6 and F3:II.1 bilateral semicircular canal (SCC) dysplasia was diagnosed by cranial MRI (Fig. 3 and Table 1), but no other cerebral anomalies were identified. Evaluation of MRI data for soft tissues and bones revealed a subtle osseous asymmetry of the skull and midface. Uni- or bilateral hearing impairment or complete sensorineural hearing loss (SNHL) was confirmed in four individuals (4/5), however, vertigo or imbalance were not reported. Motor development of the children was markedly delayed. Individual F4:II.2 did not achieve unsupported walking. All affected individuals had a mild to severe cognitive impairment. Five of them showed delayed or minimal speech development and two individuals did not develop expressive language. Individuals F1:II.6 and F5A:II.3 exhibited behavioural abnormalities with autistic features, and Individual F1:II.6 developed excessive hyperphagia. Laboratory findings were unremarkable besides transiently elevated transaminases in Individual F1:II.6.

### GestaltMatcher analysis

To validate the clinical finding of facial asymmetry in ZSCAN10 deficiency, we used the GestaltMatcher approach, an encoder for portraits.^25^ This analysis recapitulated the facial asymmetry as a clinical feature in all individuals. We observed that the subjects with ZSCAN10 deficiency were not highly similar to each other, except Individuals F2:II.2 and F2:II.3, who are siblings (Fig. 3). Comparing subjects with ZSCAN10 deficiency with other genetic disorders associated with string interactors of ZSCAN10 (*PBX1*: OMIM #617641; *SALL4*: OMIM #607323; and *SMAD4*: OMIM #139210) or suggested interacting factors (*CHD7*: OMIM #214800*)*^37^ (Supplementary Table 2), the clusters of individuals harbouring pathogenic variants in *ZSCAN10* and *CHD7* partially overlapped (Fig. 3). Individuals F1:II.6 and F3:II.1 showed a high degree of similarity and Individual F5b:II.1 also showed a certain degree of similarity to individuals with *CHD7-*associated CHARGE syndrome. This finding is consistent with the initial assessment of clinicians who considered CHARGE syndrome as a differential diagnosis. We then hypothesized that the similarity between *ZSCAN10* and *CHD7* might result from the phenotypic feature of facial asymmetry. To investigate this aspect further, we designed an experiment for this particular phenotypic feature (Supplementary material). After training the model, we tested it on the validation set of 50 images and six images of subjects with ZSCAN10 deficiency. The classification accuracy of the validation set was 82% (41/50 correct), indicating that the network learned to recognize the phenotypic feature of facial asymmetry. Moreover, all six *ZSCAN10* frontal images were correctly classified.

### *Zscan10* knockout mouse model

**Figure 4:**
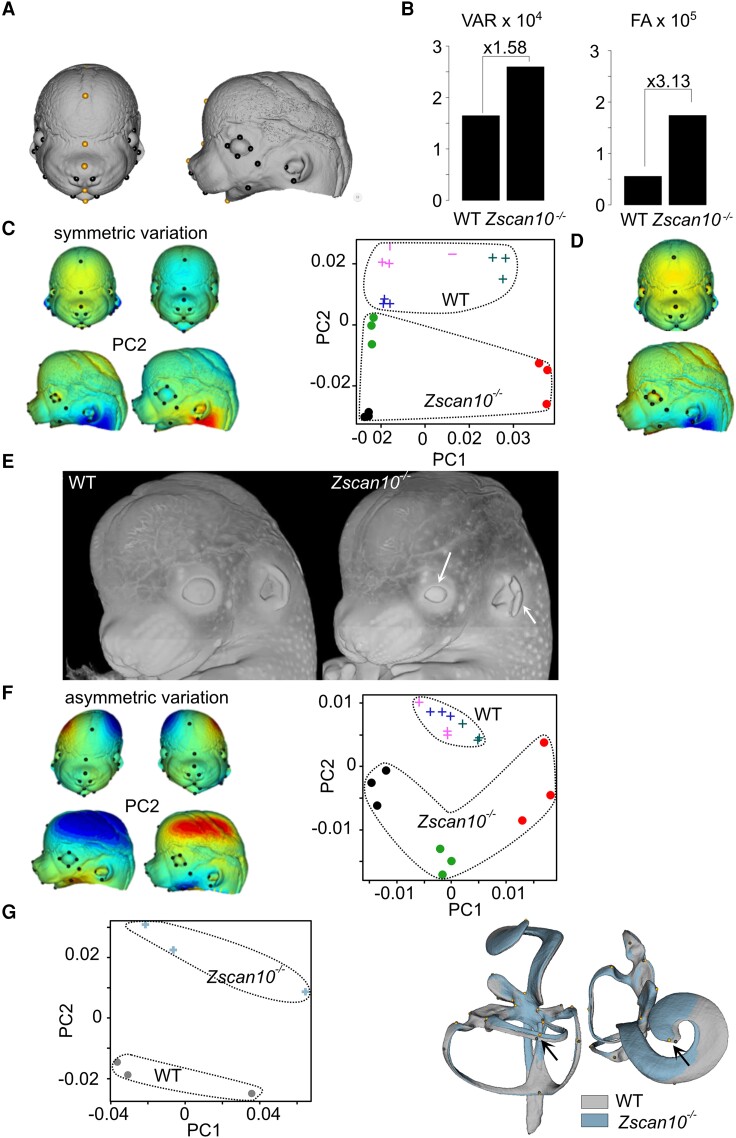
**Facial dysmorphism in *Zscan10*-deficient mouse embryos.** (**A**) Twenty-six surface landmarks (10 paired in black plus six unpaired in orange) were affixed in triplicates on three independent wild-type (WT) and three *Zscan10^−/−^* embryonic Day (E)14.5 embryos. (**B**) Among-individual Procrustes variance^32^ and fluctuating asymmetry (FA). Variance (VAR) estimates were multiplied by 10^4^ and FA by 10^5^. (**C**) Principal components of symmetric variation. *Left*: Coloured meshes correspond to variation (negative–positive) along PC1 (*left*) or PC2 (*right*). Blue corresponds to contraction and red to expansion compared with the opposite side, i.e. the shape on left corner corresponds to the shape inferred for the negative PC2 and indicates the deviation from the shape on its right; for instance, in the knockout (KO) there is a relative opening of the ear compared with the WT. *Right*: Colours correspond to individuals (three replicates per individual): filled circles represent KO individuals and crosses represent WT individuals. (**D**) Differences between symmetric averages. Shape corresponds to the least-squared (LS) means of the KO and the colours show the expansion^38^ or contraction (blue) from the WT. Shape changes are very similar to PC2 (α = 18°) from **C**. (**E**) Representative head volumes for WT and *Zscan10^−/−^* E14.5 embryos showing smaller eye size and ear opening (white arrows). (**F**) Principal components of asymmetric variation indicate that *Zscan10*-deficient and WT mice appear to have very different asymmetry patterns, with *Zscan10*-deficient mice displaying larger and more fluctuating deviations. (**G**) Principal components of inner ear reconstitution. Average reconstitutions were computed and overlayed (grey for WT and blue for *Zscan10^−/−^*) showing a mild difference between *Zscan10*-deficient and WT mice with PC2 accounting for 20% of the variance corresponding to a misalignment of one of the semicircular tubes and a shortening of the cochlea (black arrow).

Guided by clinical findings in human, we performed expanded imaging analyses to study the involvement of *Zscan10* in murine embryonic development. We used mice homozygous for a gene targeting the *Zscan10* tm2a allele (*Zscan10^tm2a(EUCOMM)Wtsi^*) (Supplementary material).^28^ We acquired 3D facial datasets from three homozygous *Zscan10-*deficient mice at embryonic Day 14.5 and three matched wild-type controls.^29^ Symmetric and asymmetric shape variations were obtained and observed differences (both within FA or between individuals) were large and significant using Fisher’s F-test (Fig. 4B). Principal component analysis (PCA) of symmetric shape variation showed that PC2, which describes skull shape, relative eye size and ear opening, consistently opposed *Zscan10*-deficient to wild-type mice (Fig. 4C–F). We next assessed the ear malformation phenotype from the generalized Procrustes analysis of 22 landmarks on the 3D inner-ear model. We detected a mild difference between *Zscan10*-deficient and wild-type mice, with PC2 accounting for 20% of the variance corresponding to a misalignment of one of the semicircular canals and a shortening of the cochlea (Fig. 4G).

## Discussion

The identification of four different bi-allelic PTVs in *ZSCAN10* in seven affected members of five families establishes *ZSCAN10* as a gene confidently implicated in this syndromic neurodevelopmental disorder. Highly consistent phenotypic features include global developmental delay, behavioural abnormalities and variable facial asymmetry with outer and inner ear malformations leading to profound SNHL. Although the expression of *ZSCAN10* decreases in the later stages of human embryonic development,^13-16^ residual expression in the brain and testis may be associated with phenotypic features that include cognitive impairment and, in some cases, microgenitalia and maldescended testis (Table 1).

*ZSCAN10* is conserved in *Boreoeutheria* with 71.21% sequence identity shared between *Homo sapiens* (NP_116194.1) and *Mus musculus* (NP_001028597.2). The human *ZSCAN10* gene has the same number of exons as the mouse orthologue, including five translated exons and one untranslated first exon. In line with the localization of the PTVs identified in affected individuals, exon 6 and thus the zinc finger C_2_H_2_ domains were targeted in the mouse model investigated in this study.^28^ In a previously published mouse model, the *Zscan10* knockout was induced by inserting a vector into intron 1 upstream of the ATG start codon.^38^ Both murine models had a C57BL/6 background and were expected to result in a complete knockout. Although they showed significant phenotypic overlap, the observed embryonic lethality in homozygous knockouts was higher in our study (Supplementary material), possibly due to dietary differences or residual amounts of ZSCAN10 derived from alternative transcripts. Owing to embryonic lethality, we cannot provide a detailed comparison to the adult mouse phenotype that was published previously.^38^ The behavioural alterations reported by Kraus *et al.*^38^ in homozygous mutant mice showed similarities to the human phenotype but comparison remains difficult. Other morphological abnormalities in this mouse model (e.g. organ and eye malformations) were not observed in our clinical cohort. Unfortunately, analysis of facial asymmetry and inner ear were not performed in adult mice.^38^ However, findings on outer and inner ear malformations as well as facial asymmetry observed in the mouse model used in this study showed striking similarities to the human phenotype of ZSCAN10 deficiency. This suggested a high degree of conservation of involved transcriptional networks between mice and humans.

To date, the exact function of ZSCAN10 is still unclear. Its interaction with SOX2 and OCT4 suggests an involvement in the pluripotency of embryonic stem cells.^12,14,39^ Therefore quantification of *ZSCAN10* transcripts has been proposed as a highly sensitive marker for the detection of undifferentiated human induced pluripotent stem cells (iPSCs), rendering this assay an approach for quality control of iPSC-derived cell therapy products.^40^ Our observations in mESCs outlined that loss of ZSCAN10 dysregulates several genes associated with pluripotency and differentiation of ESCs. Nevertheless, there is evidence suggesting that ZSCAN10 might be dispensable for pluripotency of mESCs.^41^

Human ZSCAN10 has been characterized as a regulator of the exosome complex and has a proposed role in regulating glutathione homeostasis in aged iPSCs.^17,42^ Recently, an upregulation of ZSCAN10 expression in glioma cells was associated with an OCT4-dependent oncogene with excessive cell proliferation, and the authors proposed a role in the Wnt/β-catenin signalling pathway.^43^ Our functional studies in mESCs carrying a representative human protein truncating variant clearly indicated a critical effect on ZSCAN10 function altering its subcellular localization and impairing DNA binding, in particular to the string interacting genes. These *in vitro* observations strongly suggested that the identified bi-allelic variants in *ZSCAN10* lead to a loss-of-function by activating the NMD pathway and/or altering the subcellular distribution of a potentially resulting truncated ZSCAN10 protein.

Using the GestaltMatcher approach, we confirmed facial asymmetry as a recognizable core feature of ZSCAN10 deficiency. This suggested that clinical diagnosis may be supported by an artificial intelligence approach. In addition, the phenotypic overlap with other molecularly defined syndromes could provide insights into shared pathomechanisms. While OCT4 and NANOG have not been implicated in human disease, heterozygous *de novo* variants in *SOX2* cause syndromic microphthalmia-3 (MCOPS3; OMIM #206900) characterized by eye malformations as well as extraocular manifestations including intellectually disability and hearing loss (Supplementary Table 4).^44^ Interestingly, while the overlap between *ZSCAN10*- and *SOX2*-associated presentations seems to be currently limited to facial asymmetry in humans, mice homozygous for a gene trap *Zscan10* allele were shown to phenocopy an eye malformation previously reported for *Sox2* hypomorphs.^38^ In addition to the confirmed ZSCAN10 interaction partners, which are established (*SOX2*) or *bona fide* candidate disease genes (*OCT4, NANOG*), gene–disease associations have also been described for other interactors of *ZSCAN10* annotated in STRING-DB. This includes the transcription factors *PBX1* (congenital anomalies of kidney and urinary tract syndrome with or without hearing loss, abnormal ears, or developmental delay; OMIM #617641), *SMAD4* (Myhre syndrome; OMIM #139210) and *SALL4* (Duane-radial ray syndrome; OMIM #607323) (Supplementary Table 4).^26^ Common features of these disorders comprise developmental delay, asymmetric facial features and organ malformations. For *PBX1-*associated disease, malformations of the outer ears have been reported, which are reminiscent of the abnormalities observed in ZSCAN10 deficiency.^45^ Notably, our investigation of *Zscan10*^−/−^ mESCs showed a dysregulation of *Sall4* outlining their string interaction. Furthermore, as also suggested by GestaltMatcher, the affected subjects share a high degree of facial similarity with *CHD7-*associated CHARGE syndrome (OMIM #214800; Fig. 3), which often comprises dysplasia of the SCCs.^46^ So far, no direct functional link between ZSCAN10 and CHD7 has been established in humans. However, it has been shown that CHD7 co-localizes with the transcription factors OCT4, SOX2 and NANOG, which are known interaction partners of ZSCAN10.^47^ In mice, Zscan10 and Chd7 are both reported to maintain pluripotency during early embryonic development as interacting factors within the same transcriptional network.^37,48^ Comparable to the *Zscan10* knockout mouse model, *Chd7* mice also show morphological abnormalities as well as embryonic lethality.^49,50^ Although further investigations using appropriate model systems are needed to define the molecular cascades that cause pathology, our study further supports the importance of ZSCAN10 in embryonic development.

## Supplementary Material

awae058_Supplementary_Data

## Data Availability

Sequence datasets have been generated and contributed by different study sites and have not been deposited in a public repository due to varying local consent regulations. Depersonalized data and additional experimental data that this study is based on, can be provided upon request. All variants have been deposited into ClinVar (https://www.ncbi.nlm.nih.gov/clinvar/) under Institute of Medical Genetics and Applied Genomics, University of Tübingen, including SUB11294153, SUB11294175, SUB11294201 and SUB11294215. RNA-seq data generated for this work have been deposited to GEO under accession number GSE234680 (token: sdghqywezbexpol).

## Web resources

The URLs for the data presented herein are as follows:

gnomAD server, https://gnomad.broadinstitute.org

The Human Protein Atlas, https://www.proteinatlas.org

GTEx Portal, https://gtexportal.org

Online Mendelian Inheritance in Man (OMIM), https://www.omim.org

Ensembl Variant Effect Predictor, https://www.ensembl.org/Homo_sapiens/Tools/VEP

Combined Annotation Dependent Depletion, https://cadd.gs.washington.edu

UCSC, https://genome.ucsc.edu (UCSC Genome Browser on Human, GRCh38/hg38 Assembly)

STRING-DB, https://string-db.org (v11.5)

NMDEscPredictor, https://nmdprediction.shinyapps.io/nmdescpredictor/
